# Effect of feed restriction and refeeding on performance and metabolism of European and Caribbean growing pigs in a tropical climate

**DOI:** 10.1038/s41598-019-41145-w

**Published:** 2019-03-19

**Authors:** Nausicaa Poullet, Jean-Christophe Bambou, Thomas Loyau, Christine Trefeu, Dalila Feuillet, David Beramice, Bruno Bocage, David Renaudeau, Jean-Luc Gourdine

**Affiliations:** 1 UR143 URZ, INRA, Petit-Bourg (Guadeloupe), F-97170 France; 2 Present Address: IDEXX Laboratories, 84 Rue Charles Michels, Saint-Denis, F-93200 France; 3 UMR1348 PEGASE, INRA Agrocampus Ouest, St Gilles, F-35590 France; 4 UE1294 PTEA, INRA, F-97170 Petit-Bourg (Guadeloupe), France

## Abstract

Reduction in feed intake is a common physiological response of growing pigs facing stressful environmental conditions. The present experiment aims to study (1) the effects of a short-term feed restriction and refeeding on pig performance and metabolism and (2) the differential response between two breeds, Large White (LW), which has been selected for high performance, and Creole (CR), which is adapted to tropical conditions. A trial of 36 castrated male pigs (18 LW and 18 CR) was carried out. For each breed, half of the animals were restrictively fed at 50% of the standard feed allowance for 6 days and then fed normally for the next 14 days. Growth performance, thermoregulatory responses, plasma hormones and metabolites were measured. Results showed that, for all traits, the difference in response between the two breeds was small and rarely significant, which may be due to the short duration of the feed restriction. Irrespective of breed, feed restriction induced a reduction of growth rate and feed efficiency that was rapidly compensated for upon refeeding. Feed restriction also reduced skin temperature, rectal temperature and respiratory rate, as well as blood urea and cholesterol, which are of interest as potential biomarkers for feed restriction.

## Introduction

Growing pigs may face periods of feed restriction due to economic reasons or environmental factors. Voluntary reduction of feed intake, which leads to feed restriction, is a common physiological response of growing pigs facing stressful environmental conditions, such as heat stress, poor sanitary conditions, social stress or disease pressure^1–3^. With the increase of worldwide animal production, especially in tropical and subtropical regions^4^, improving livestock systems efficiency and animal welfare in response to stressful conditions becomes a crucial issue. A better understanding of the impact of feed restriction on pig physiology and metabolism would allow to set up strategies to limit the economic impact of stressful conditions and improve animal welfare during stress. During feed restriction, the growing pig must adjust its metabolism to maintain homeostasis through changes in nutrient partitioning between growth and maintenance. The animal responses to feed restriction is highly variable within and between populations and part of this variability may have a genetic basis^5,6^. There is a crucial need of information on local breeds and on their adaptation to specific environmental conditions, as they constitute genetic resources that are essential to maintain livestock systems efficiency in the context of climate change^7^. The Creole pig breed (CR) is the most important Caribbean local breed both in population size and economic importance. The CR has not been submitted to genetic selection and is characterized by early maturity, increased fat deposition and a good adaptation to harsh environmental conditions, including feed restriction periods^8,9^. Therefore, The CR provides a good model to study the genetic variability in the response to feed restriction in pigs by comparing it to the Large White breed (LW) that has been selected for high growth performance in optimal conditions. The aims of the present experiment were (1) to study the effects of a short-term drastic feed restriction and subsequent refeeding on pig postabsorptive metabolism and (2) to compare the response of two breeds differing in their growth potential to feed restriction, LW and CR. We hypothesized that feed restriction would affect pig growth performance, thermoregulation and metabolism differently in the two breeds.

## Results

### Climatic characteristics

**Figure 1:**
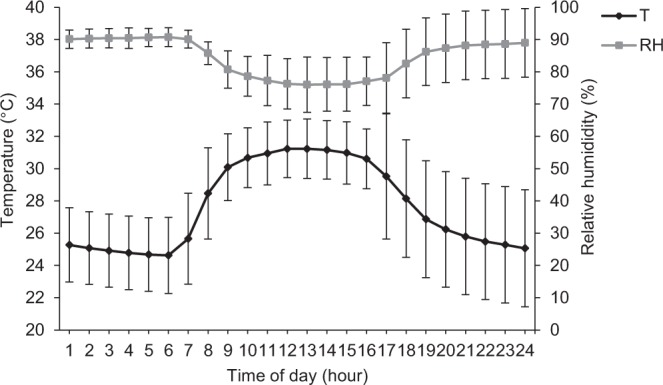
Daily climatic fluctuation of average ambient temperature (T – black line) and average relative humidity (RH – grey line) in the pig building facility. Error bars represent standard deviation.

Figure 1 shows the variation of hourly ambient temperature and relative humidity in the experimental facility. The average ambient temperature and relative humidity during the trial were 27.4 °C and 84.0%, respectively. The daily ambient temperature ranged from 24.4 to 30.1 °C. The relative humidity varied between 74.1 and 90.8%. The hourly variation in ambient temperature (+27% between lowest and highest values) was higher than in relative humidity (+19% between lowest and highest values). The hourly fluctuation of ambient temperature showed that the minimum and maximum values were reached at 0600 h (24.6 °C) and at 1300 h (31.2 °C). Contrary to ambient temperature, relative humidity was greatest at about 0600 h and lowest at about 1300 h (i.e., 90.7 and 76%, respectively).

### Effects on growth performance

Effect of treatment and period on growth performance parameters are presented in Table 1. As anticipated, ADFI was similar (on average 1.86 kg/d, P > 0.1) during P1 and P3 for both treatments. There was a positive residual correlation between ADFI and ADG (r = 0.33, P < 0.01, Table 2). During feed restriction in P2, growth performance was negatively affected. Irrespective of breed, during P2, RF pigs had lower ADG (−75%, P < 0.001) and were less efficient than NF pigs (0.43 vs. 0.19, P < 0.05). On the contrary, during the refeeding period P3, ADG was increased in RF pigs compared to NF pigs (+25%, P < 0.001). Average daily feed intake being similar (P = 0.91) between RF and NF pigs during the refeeding period due to the experiment design, RF animals had higher FE than NF pigs during refeeding (+49%, P < 0.001). Performance for the total experimental period (including feed restriction) was also affected by feed restriction. Average daily feed intake and ADG were reduced for RF pigs compared to NF pigs (−13%, P < 0.001 and −16%, P < 0.001, respectively for ADFI and ADG). However, FE for the total experiment period did not differ between treatments (P = 0.42). Similarly, there was no significant difference (P = 0.22) between treatments in BFG over the total experimental period. Overall, there was no effect of treatment (P = 0.73) or treatment x period interaction (P = 0.10) on BFT, but we found a positive residual correlation between BFT and ADFI (r = 0.25, P < 0.05, Table 2).

**Table 1:** Effect of treatment and period on growth performance.

Item	Normal Feeding	Restricted Feeding	RSD^1^	Significant effect^2^
Number of animals	18	18		
**Final BW** ^**3**^ **, kg**				
d0	45.1	46.9	1.00	P***, PxT***, BxP***
P1	47.3	49.1		
P2	52.8	50.3		
P3	60.0	59.4		
**ADFI** ^**4**^ **, kg/d**				
P1	1.86^a^	1.83^a^	0.03	T***, P***, PxT***
P2	1.91^a^	0.98^b^		
P3	1.85^a^	1.87^a^		
Total test period	1.87^a^	1.64^b^	1.81	R^†^, T***
**ADG** ^**5**^ **, g/d**				
P1	536.5^bc^	549.1^bc^	1.11	R***, T**, B***, PxT***
P2	823.2^a^	204.0^d^		
P3	520.0^c^	648.5^b^		
Total test period	604.3^a^	507.6^b^	61.4	T***, B***, BxT^†^
**BFT** ^**6**^ **, mm**				
P1	10.1	10.3	2.08	R***,B***, P***, BxP***, PxT^†^
P2	11.2	11.4		
P3	14.2	13.1		
**BFG/BFT**_**P1**_^**7**^				
Total test period	0.39	0.31	0.18	R*
**FE** ^**8**^ **, kg of gain/kg of feed**				
P1	0.29^b^	0.29^b^	0.08	R***, PxT***
P2	0.43^a^	0.19^b^		
P3	0.28^b^	0.40^a^		
Total test period	0.33	0.31	0.03	R***, B***

^a–d^Within a period, means with a different superscript letter differ, P < 0.05.

^1^Residual Standard Deviation.

^2^From an analysis of variance with a linear mixed model including the effects of Treatment (T), Breed (B), Period (P), Replicate (R) and their interactions as fixed effect. Statistical significance: ***P < 0.001, **P < 0.01, *P < 0.05, ^†^P ≤ 0.10.

^3^BW = Body Weight.

^4^ADFI = Average Daily Feed Intake.

^5^ADG = Average Daily Gain.

^6^BFT = Backfat Thickness.

^7^BFG/BFT_P1_ = Backfat Gain/initial BFT (in P1).

^8^FE = Feed Efficiency.

**Table 2:** Pearson residual correlation coefficients for hormones and performance traits.

Trait	Ghrelin	Leptin	Insulin	BFT^a^	ADG^b^	ADFI^c^
Ghrelin	—					
Leptin	−0.09	—				
Insulin	−0.29**	−0.1	—			
BFT^a^	−0.07	0.16	−0.03	—		
ADG^b^	−0.30*	0.17	−0.004	−0.01	—	
ADFI^c^	0.22	−0.06	0.25*	0.25*	0.33*	—

^a^BFT = Backfat Thickness.

^b^ADG = Average Daily Gain.

^c^ADFI = Average Daily Feed Intake.

Statistical significance: ***P < 0.001, **P < 0.01, *P < 0.05, ^†^P ≤ 0.10.

**Figure 2:**
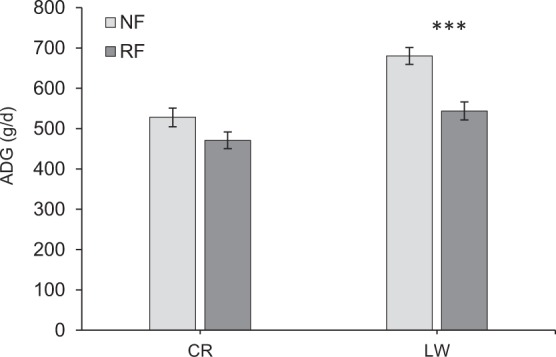
Average Daily Gain (ADG) over the total experimental period in Creole (CR) and Large White (LW) pigs under Normal Feeding (NF) or Restricted Feeding (RF) treatment. Data are reported as least squares means and error bars represent standard error. Asterisks indicate significant differences ***P < 0.001, **P < 0.01, *P < 0.05.

No interaction between breed and treatment was found for any performance trait (P > 0.05). Nevertheless, there was a tendency for a breed × treatment effect (P = 0.06) for ADG over the total experimental period. Average daily gain in CR pigs was similar between treatments (500 g/d on average, P = 0.25) whereas ADG in LW pigs was lower in RF than in NF (544 g/d vs. 680 g/d, P < 0.001) (Fig. 2). Irrespective of treatment or period, LW pigs had greater ADG compared to CR pigs (606 g/d vs. 487 g/d, P < 0.001) even though they had similar ADFI (1.71 kg/day on average, P = 0.9). Consequently, LW pigs were more efficient than CR pigs (0.33 vs. 0.28, P < 0.001). Irrespective of treatment, BFT was higher in CR than in LW (15.1 mm vs. 8.3 mm, P < 0.001). However, there was no difference in backfat accumulation between the two breeds over the total experimental period, as BFG corrected for initial BFT (in P1) was not significantly different between the two breeds (P = 0.19).

### Effects on thermoregulatory responses

Effects of treatment and period on thermoregulatory responses are presented in Table 3. For all thermoregulatory body measures (RR, ST, RT), we found a significant treatment x period interaction (P < 0.05). For all three parameters, a significant difference between treatments was observed only during the restriction period P2. During P2, RF pigs had a reduced RR, ST and RT (P < 0.01) compared to NF animals (−13 bpm, −0.7 °C, −0.4 °C, respectively for RR, ST and RT). No effect of breed or interaction between breed and treatment (P > 0.05) was found for RR or ST. However, there was a breed × treatment interaction for RT (P < 0.05), as RT was higher in LW pigs than in CR pigs in the RF treatment (39.5 °C vs. 39.1 °C, P < 0.05) whereas RT was similar between breeds in NF pigs (39.3 °C, 39.4 °C, respectively in LW and CR, P = 0.62). Irrespective of treatment or period, ST was lower in CR pigs compared to LW (37.0 °C vs. 37.6 °C, P < 0.001).

**Table 3:** Effect of treatment and period on thermoregulatory parameters.

Item	Normal Feeding	Restricted Feeding	RSD^1^	Significant effect^2^
Number of animals	18	18		
**Rectal Temperature, °C**				
P1	39.4^a^	39.5^a^	0.66	P***, H***, BxT*, PxT**
P2	39.3^a^	38.8^b^		
P3	39.3^a^	39.5^a^		
**Skin Temperature, °C**				
P1	37.3^a^	37.2^a^	0.96	R***, H***, B***, P*, PxT*
P2	37.8^b^	37.1^a^		
P3	37.1^a^	37.2^a^		
**Respiratory Rate, bpm**				
P1	64.2^a^	59.2^a^	19.3	R***, H***, P**, PxT***
P2	67.1^a^	54.5^b^		
P3	66.7^a^	73.3^a^		

^a–c^Within a period, means with a different superscript letter differ, P < 0.05.

^1^Residual Standard Deviation.

^2^From an analysis of variance with a linear mixed model including the effects of Treatment (T), Breed (B), Period (P), Hour (H), Replicate (R), and their interactions as fixed effect. Statistical significance: ***P < 0.001, **P < 0.01, *P < 0.05, ^†^P ≤ 0.10.

### Effect on pig behaviour and activities

Table 4 show the effects of feed restriction on pig postures and activities. Normally-fed pigs spent more time lying on their side (73% vs. 65% of the 24h-period, P < 0.01) and less time lying on their belly (14% vs. 25% of the 24h-period, P < 0.001), compared to RF pigs. Normally-fed animals also spent more time standing (7.8% vs. 6.2% of the 24h-period, P < 0.05) and more time eating compared to RF pigs (7.1% vs. 3.8% of the 24h-period, P < 0.001). We found no effect of treatment on time spent drinking (P = 0.32). No breed x treatment interaction was found for pig postures or activities. Irrespective of treatment, CR pigs spent more time eating (6.5% vs. 4.6% of the 24h-period, P < 0.01) and were more active [more time standing (7.9% vs. 6.1% of the 24h-period, P < 0.05) and less time sitting (3.3% vs. 5.7%, P < 0.01)] than LW animals.

**Table 4:** Effect of treatment on pig behavior and activity.

Item^a^	Normal Feeding	Restricted Feeding	RSD^b^	Significant effect^c^
Number of animals	12	12		
Sternal Lying	14.3	24.6	6.2	R*, T***
Lateral Lying	73.2	64.9	7.0	R*, T***
Sitting	4.7	4.3	2.0	B**
Standing	7.8	6.2	1.9	B*, T*
Eating	7.1	3.8	1.4	R**, B**, T***
Drinking	3.0	2.3	1.5	

^a^Postures and activities are expressed as percent of time spent in the posture or activity over the 24 h scanning period.

^b^Residual Standard Deviation.

^c^From an analysis of variance with a linear mixed model including the effects of Treatment (T), Breed (B), Period (P), Replicate (R) and their interactions as fixed effect. Statistical significance: ***P < 0.001, **P < 0.01, *P < 0.05, ^†^P ≤ 0.10.

### Effects on plasma hormones and metabolites

Effects of treatment and period on plasma hormones and metabolites are presented in Table 5. No period x treatment interaction was found for any plasma hormones (P > 0.1) but we found a negative residual correlation between ghrelin and insulin (r = −0.29, P < 0.01, Table 2). There was also a negative residual correlation between ghrelin and ADG (r = −0.30, P < 0.05, Table 2) and a positive correlation between insulin and ADFI (r = 0.25, P < 0.05, Table 2). Irrespective of treatment or period, CR pigs had higher plasma insulin (8.4 µU/mL vs. 6.2 µU/mL, P < 0.05) and higher leptin levels than LW pigs (14.4 ng/mL vs. 6.3 ng/mL, P < 0.001). On the contrary, we found no effect of breed or interactions between breed and treatment for plasma ghrelin (P > 0.1).

**Table 5:** Effect of treatment and period on plasma hormones and metabolites.

Item	Normal Feeding			Restricted Feeding			RSD^1^	Significant effect^2^
	P1	P2	P3	P1	P2	P3		
Number of animals	18	18	18	18	18	18		
Insulin, µUI/ml	8.00	10.27	8.36	4.29	4.46	8.37	0.98	R***, T**, B*
Ghrelin, pg/mL	248.5	191.2	204.2	317.3	223.0	238.0	1.00	T*, P*, BxP**
Leptin, ng/mL	14.34	7.48	12.10	13.36	5.96	10.25	0.98	B***, P***, BxP**
ALK Phos^3^, U/L	38.8	37.0	31.2	35.3	27.2	32.1	0.99	R***, P*, BxP*, PxT^†^
GGT^4^, U/L	35.2	33.2	36.8	33.4	33.2	32.1	0.99	R***
AST^5^, U/L	29.8	34.2	39.5	27.6	32.1	29.5	1.01	
ALT^6^, U/L	43.0	42.5	39.4	43.9	46.0	34.8	0.99	P*
Amylase, U/L	999	1036	1058	1006	1058	956	0.96	R^†^, B*, BxP^†^, PxT*
BUN^7^, g/L	0.28^ab^	0.28^ab^	0.32^a^	0.26^b^	0.19^c^	0.29^ab^	0.10	B***, T*, P***, PxT*
Glucose, g/L	0.91	0.89	0.90	0.90	0.86	0.89	0.09	R**
Phosphorus, mg/L	68.4	67.8	61.3	69.0	77.1	71.6	1.00	R***, T*
Albumin, g/L	34.5	35.0	36.0	35.2	36.4	33.5	1.24	PxT*
Cholesterol, g/L	0.71^ab^	0.74^a^	0.76^a^	0.73^a^	0.63^b^	0.60^b^	0.10	R*, T*, B^†^, PxT***
Creatinin, mg/L	11.9	12.7	13.8	11.7	12.7	12.9	1.10	R**, P***, B***
Total protein, g/L	63.4	62.1	63.3	64.9	65.4	67.4	0.98	T**, B***
Globulines, g/L	29.0	27.0	27.3	29.4	28.6	33.8	4.76	T*, B*, PxT^†^

^a–c^Within a period, means with a different superscript letter differ, P < 0.05.

^1^Residual Standard Deviation.

^2^From an analysis of variance with a linear mixed model including the effects of Treatment (T), Breed (B), Period (P), Replicate (R) and their interactions as fixed effect. Statistical significance: ***P < 0.001, **P < 0.01, *P < 0.05, ^†^P ≤ 0.10.

^3^Alkaline Phosphatase.

^4^Gamma γ-glutamyl transferase.

^5^Aspartate Transaminase.

^6^Alanine Transaminase.

^7^Blood Urea Nitrogen.

Concerning blood metabolites, irrespective of breed, BUN and cholesterol were reduced during the feed restriction period P2 (−49% and −18% respectively, P < 0.05). Levels of BUN did not differ between RF and NF animals before and after feed restriction (P1 and P3, P > 0.05). However, levels of cholesterol were similar between RF and NF before feed restriction (P1, P = 0.78) but were reduced in RF animals compared to NF animals after refeeding (P3, −26%, P < 0.05). There was no treatment or treatment x period interaction for GGT, ALT, AST, creatinine and glucose (P > 0.05).

There was no significant breed × treatment interaction for any of the plasma metabolites measured (P > 0.05). Irrespective of treatment or period, CR pigs had lower creatinine, total protein levels and globulins compared to LW animals (11.5 vs. 13.7 mg/L, P < 0.001; 61.9 vs. 67.0 g/L, P < 0.001; 27.3 vs 31.0 g/L, P < 0.05, respectively,). On the contrary, CR pigs had higher BUN concentration than LW pigs (0.31 vs. 0.23 g/L, P < 0.001).

## Discussion

Short periods of feed restriction may occur during pig growth due to economic reasons or external factors, such as heat waves, inflammatory stress, feed transition or social stress^3^. In the present study, we aimed at understanding the effects of a short and drastic feed restriction on pig metabolism. As observed in this study, it is well established that feed restriction induces a reduction in growth rate in growing pigs^10–13^. However, depending on the severity of the feed restriction, the reduction of feed intake does not necessarily translate into a proportional reduction in growth rate. Two opposing mechanisms affect weight gain during feed restriction. On one hand, nutrient availability for growth is reduced and a greater part of the metabolizable energy is used for maintenance^11,14,15^ and on the other hand, a greater fraction of the retained energy is retained as protein, rather than lipid^11,16^. Due to the association of water with protein, the reduction in ADG may be less than proportional when feed restriction is not too severe, leading to similar feed efficiency between restricted and normally fed pigs^12,16^. However, in the case of a drastic feed restriction (as in this experiment), it is expected that the reduction in ADG will be greater than the reduction in ADFI, due to the increased relative importance of maintenance^11^. Indeed, we observed a reduced FE during feed restriction (−55%), resulting from a greater reduction in ADG than in ADFI (−75% and 50% respectively). Consistent with these results, we also found a positive residual correlation between ADG and ADFI. The reduction in physical activity observed during feed restriction, as RF pigs spent less time standing compared to NF pigs (−25%), can be partly explained by the reduced time they spent eating (−47%) due to their reduced feed allowance and would have a negligible impact on their maintenance needs^17^.

The reduction of growth rate and feed efficiency during feed restriction suggests changes in pig metabolism and nutrient utilization. Therefore, modifications in blood metabolites and hormones levels could be expected. A meta-analysis of publications in different species (cattle, sheep, goats, horses, pigs, and rats) show that BUN levels can be used to predict relative differences in urinary N excretion for animals of a same type and of a same stage of production^18^. In the present study, pigs fed a restricted diet had reduced BUN levels, which could be due to a reduction in urinary N loss during feed restriction. Feed restriction in growing pigs is known to reduce faecal and urinary N excretion^11,19^. In cattle, BUN levels have also been associated with protein intake^20^, which would be consistent with a lower BUN level during feed restriction. In addition, in the present study, cholesterol levels were also reduced during feed restriction. This is consistent with previous studies in chicken showing a reduction of cholesterol during feed restriction^21^.

Leptin and ghrelin are hormones involved in the regulation of appetite and food intake in several species^22,23^, including pigs^24–26^. Ghrelin is secreted by the stomach in response to hunger or starvation, circulates in the blood, informing the central nervous system to stimulate feeding^23^. Higher plasma ghrelin were reported after fasting in humans^27^ and rodents^28^. In growing pigs, previous studies demonstrated that ghrelin was aligned with energy balance^29^ and was negatively correlated to ADG^30^. In the present study, we observed a negative residual correlation between plasma ghrelin and ADG, suggesting that circulating ghrelin may reflect the animal energy balance. However, there was no effect of feed restriction on plasma ghrelin. Salfen *et al*.^26^ reported a significant ghrelin increase after 36–48 h fasting in weaning piglets. Similarly, Govoni *et al*.^24^ showed increased ghrelin after 3-d fasting in prepuberal gilts. But consistent with our results, feed restriction at 50% of the ad-libitum level failed to affect ghrelin levels in growing pigs^31^, suggesting that feed restriction up to 50% is not sufficient to induce ghrelin response in growing pigs. Leptin, which is an appetite-suppressor hormone secreted by adipocytes is the counteracting hormone of ghrelin^22,23^. Leptin levels decrease during fasting in mice^32^, cattle^33^ and human^34^. Similarly, fasting induced reduction of plasma leptin in growing pigs^25^, gilts^35^, and weanling piglets^26^. However, feed restriction failed to affect leptin concentration in feed-restricted gilts (33% of maintenance)^36^ and long-fasting (more than 24 h) was necessary to reduce leptin mRNA expression in growing pigs^37^. These results show that a drastic and long feed deprivation is needed to reduce leptin levels in pigs. Consistent with these results, in the present study, no difference in plasma leptin was found between treatments. Plasma leptin levels have been proposed as an effective physiological regulator of fat deposition in pigs^38–40^. However, no residual correlation was found between leptin and BFT in the present study. Concerning BFT, there was no effect of feed restriction and BFT did not differ between the two groups during the refeeding period either. Nevertheless, there was a positive residual correlation between ADFI and BFT. Feed restriction induces a coordinate decrease of tissue turnover and affects orderly viscera, fat and then muscle^15^ and has been shown to induce a reduction in fat deposition in growing pigs^41,42^ and in other livestock species^43,44^. In our experiment, the late onset (at 45 kg of BW) and short duration of the restriction period (7d) could explain the absence of an effect of feed restriction on BFT, as the period of feed restriction used in other studies occurred earlier in life and/or was longer (from 20–45 kg^41^, from weaning to 50–80 kg of BW^42^).

In contrast to our expectations, insulin and glucose levels did not decrease during feed restriction. Previous studies in growing pigs^31,45^ and in other species^46^ showed that plasma insulin concentration decreased during feed restriction. This discrepancy with the literature could be explained by the short duration of feed restriction before blood sampling, as blood was sampled four days after the beginning of the RF treatment. Nevertheless, we found a positive residual correlation between insulin and ADFI, suggesting that insulin is related to the level of feed intake. On the contrary, we found a negative residual correlation between ghrelin and insulin, suggesting that they are secreted in an opposite manner. Insulin inhibits ghrelin secretion in humans^47^ and insulin and ghrelin were negatively correlated in pigs that were fed one meal a day (which was also the case in our study)^48^. Concerning blood glucose, levels are markedly resistant to prolonged feed deprivation in chickens^21,49^ whereas in pigs, blood glucose levels sensitivity depends on the severity of feed restriction. Complete fasting induces a reduction of blood glucose in prepuberal gilts^35^ whereas no change in blood glucose is observed upon dietary or protein restriction in pregnant gilts^50^. The absence of change in insulin and glucose levels during feed restriction may explain that leptin levels were not affected by feed restriction either, as insulin and glucose can modulate leptin gene expression and secretion^25,51,52^.

In order to evaluate the effect of feed restriction on thermoregulation, different thermoregulatory parameters (ST, RR and RT) were measured. We found that our feed restriction protocol resulted in a reduction of all thermoregulatory measures. This result could be explained by a decrease in heat production due to lower feed intake and thus lower feed digestion^53^. A reduction of RT of a similar magnitude (−0.4 °C) during feed restriction in growing pigs has been observed in other studies^45,54,55^. Cervantes *et al*.^56^ observed a reduction in body temperature (tympanic temperature) and respiratory rate in pigs fed a restricted diet compared to ad libitum-fed pigs. In the present study, unlike most feed restriction studies where animals were raised at thermoneutral conditions, animals were raised indoor at an environmental temperature (27.4 °C) above the thermoneutral zone (around 25 °C for growing pigs)^57^. This heat stress resulted in hyperthermia in all body temperature measures. Therefore, the high values of ST, RR and RT in NF animals could explain the reduction of all thermoregulatory parameters during feed restriction. Pig postures during feed restriction were consistent with thermoregulatory measures. Normally-fed pigs spent more time lying on their side than RF pigs, suggesting that they chose lateral rather than sternal lying to dissipate the excess heat generated by their increased feed allowance compared to RF pigs. Previous studies showed that heat-stressed pigs tend to lie preferentially on their side to reduce heat^58^.

After periods of feed restriction, animals may exhibit a period of accelerated growth, referred to as compensatory growth^15^. Compensatory growth in pigs depends on the onset, severity and duration of the restriction period and the onset and duration of refeeding^11,12,59,60^. We confirmed that the length of the refeeding period is an important parameter to observe compensatory growth, as we observe a significant increase in ADG in RF pigs after the 14-day refeeding period whereas, with a similar design but shorter refeeding period (7 days), Lovatto *et al*.^11^ did not observed compensatory growth. In the current study, ADG over the whole experimental period (including feed restriction) was lower in RF than in NF, suggesting that compensatory growth was not able to restore similar ADG in RF pigs over the total experimental period. However, it is important to note that our experiment design included a controlled feed intake during the normal feeding periods, resulting in lower ADFI in RF than in NF pigs over the whole experimental period, which may have limited the extent of compensatory growth^11,59^. Nevertheless, we found that with similar ADFI between treatments and higher ADG during refeeding, RF pigs were more efficient than NF pigs during the refeeding period. This increased efficiency could be due to a lower metabolic rate linked to reduced weight of viscera during the initial phase of compensatory growth^15,53^. In cattle, increased efficiency during refeeding is attributed to lower maintenance energy requirements concomitant with a higher proportion of tissue gain in the form of protein^44,61^. Increased efficiency during compensatory growth could also be due to a more efficient utilization of nutrient and energy after feed restriction compared to normally fed pigs. Lovatto *et al*.^11^ tested this hypothesis and found no difference in metabolic energy utilization and heat partitioning between restricted and normally fed growing pigs. However, their short refeeding period (7 days) may have limited the potential to observe compensatory mechanisms in energy utilization.

Two breeds were compared in this study: the European LW breed that have been selected for high growth performance and high lean deposition rate in optimal growing conditions, and the Caribbean CR breed, characterized by high fat deposition and a good adaptation to harsh environmental conditions^8,9^. Little information is available on the post-absorptive metabolism of CR pigs and how it relates to their performance. Irrespective of treatment, CR pigs had similar ADFI but lower ADG than LW pigs, consistent with previous results obtained at our research station^8,62^. Consequently, as observed in previous studies^8^, CR pigs were less efficient than LW pigs. This low FE could be associated with the high fat deposition rate of CR pigs. Similar conclusions were reported when Meishan pigs were compared to traditionally lean pigs^63^. Indeed, as previously demonstrated^8,9^, CR pigs had higher fat content (BFT) than LW. The low FE of CR pigs could also be due to lower N utilization efficiency. In the present study, we observed increased BUN levels in CR pigs compared to LW pigs, suggesting increased urinary N excretion^18^ and thus potential lower N utilization efficiency. In addition, we found that total protein levels were lower in CR pigs than LW. Total proteins include two major kinds of proteins, albumin and globulins. As albumine levels were similar between the two breeds, a lower level of globulin in CR pigs was responsible for the lower total protein level in CR.

The increased propensity for fat deposition in CR pigs could be associated with their feeding behaviour, as they have lower number of meals counterbalanced by higher meal size and longer ingestion rate^8^. Consistent with these results, in the current study, CR were more active and spent more time eating than LW. Ghrelin levels are altered by feeding pattern, as ghrelin is elevated in meal-fed pigs compared to ad libitum-fed pigs^29,48^. However, in the current study, no effect of breed was found for plasma ghrelin. This could be due to the distribution of the meal, which was offered once per day and did not allow a clear differentiation of feeding patterns between the two breeds.

Despite the absence of significant residual correlation between leptin and BFT, CR pigs had higher leptin than LW. This positive association between leptin and BFT has been demonstrated before^38–40^. We also found that CR pigs had higher insulin levels than LW pigs, consistent with previous studies on lean and obese pigs^64^. On the contrary, creatinine, which is a useful indicator of total body mass of striated muscle in human and dog^65^, was reduced in CR pigs compared to LW. In pigs, a study aiming at predicting phenotypes from metabolomics data showed that creatinine was positively correlated with lean meat percentage^66^. No effect of breed was found for ghrelin levels, showing that the two breeds do not differ in their basal levels of ghrelin.

CR pigs have been shown to be more tolerant to heat than LW pigs^62,67^. Consistent with this result, we found that irrespective of treatment, CR had lower ST than LW, suggesting that CR pigs produce less heat. However, no difference of breed was found for RT or RR. Renaudeau *et al*.^8^ observed an effect of breed on RR and RT above an ambient temperature of 30 °C, which was not the case in the present study (on average 27.4 °C).

Based on previous work on heat tolerance^62,67^ and on the positive association between environmental sensitivity and selection for high levels of production^68,69^, we hypothesized that LW pigs would be less tolerant to feed restriction than CR pigs. However, we found no effect of breed on growth performance during feed restriction and subsequent refeeding. This result could be a consequence of the imposed feed intake during refeeding, which may have limited the extent of compensatory response in CR pigs. Nevertheless, there was a tendency for a breed x treatment effect for ADG over the total experimental period, suggesting that overall CR pigs may be more tolerant to feed restriction than LW pigs. This tendency for increased tolerance of CR to feed restriction compared to LW could originate from their increased fat deposition which may allow dynamical storage of energy that could be mobilized during feed restriction. High producing pigs in thermoneutral conditions were less robust to heat stress and thus to a certain extend to feed restriction, than those with low growth in thermoneutral conditions^70^. Moreover, when comparing two strains of pigs differing in their lean content, Hogberg and Zimmerman^6^ found that growth rate was more reduced in lean-strain than in fat-strain during protein restriction and that upon refeeding, the fat-strain made partial or complete compensation which was not the case for the lean-strain. However, De Greef *et al*.^16^ found conflicting results and observed no difference in growth performance during protein deficiency and refeeding between two strains of pigs differing in lean contents. Similarly, Mason *et al*.^42^ compared the effect of restricted feeding in Duroc and Landrace pigs and found that breed did not affect performance during feed restriction.

Consistent with the few differences in performance parameters between the two breeds in response to feed restriction, we also found few breed x treatment effects for thermoregulation, plasma hormones and metabolites. We found a significant breed x treatment effect only for RT, as LW pigs had higher RT than CR in the RF treatment. The difference could be due to the age difference between LW and CR (3 weeks younger for LW pigs). Indeed, Rose *et al*.^71^ reported higher RT in pigs at 19 weeks of age than at 21 or 23 weeks of age, either due to an acclimation of the animals to heat stress or to a lower heat production with age due to differences in lean and fat tissues deposition rates with age.

In conclusion, the present study demonstrates that a short period of drastic feed restriction during pig growth, despite an immediate effect on growth rate, can be rapidly compensated for when normal feed allowance is resumed. Feed restriction significantly reduced all thermoregulation parameters, confirming that feed restriction reduces heat production. Our feed restriction protocol had few effects on the blood hormones and metabolites measured. Nevertheless, certain blood metabolites, such as BUN and cholesterol were very sensitive to feed restriction and should be investigated further for their use as biomarkers. The difference in response between the two breeds was small and rarely significant for all measured traits. However, further studies with longer feed restriction would be needed to investigate how genotype may affect environmental sensitivity.

## Methods

All measurements and observations on animals were performed in accordance with the current law on animal experimentation and ethics (#69-2016-1 from the Animal Care and Use Committee of French West Indies and Guyana) and the experimental protocol was approved by the French Ministry of Agriculture and Fisheries (#A971-18-02) under the direction of J. Fleury (INRA-PTEA).

### Animals and experiment design

A total of 36 barrows (18 LW and 18 CR) were used in 3 replicates on the experimental facilities of INRA in Guadeloupe, French West Indies. For each replicate, six LW and six CR pigs originating respectively from the same litter were used. In order to allow comparison of both breeds at similar Body Weight (BW), the experiments began at 16 and 19 weeks of age for LW and CR pigs respectively, allowing a similar average BW at the beginning of the experiment (d0) for both breeds (46.7 ± 1.3 kg and 45.3 ± 1.1 kg for LW and CR, respectively). Such a design was used to take into account the marked difference in Average Daily Gain (ADG) between CR and LW pigs^8^.

Pigs were housed in individual metal-slatted pens (0.85 × 1.50 m) equipped with a stainless steel feeder and a nipple water drinker. Animals could see, smell and hear each other to maintain a maximum of social interactions. All animals were adapted to the pens for thirteen days before the start of the experiment. Animals were fed a conventional diet as pellet, formulated to meet the nutritional requirements of LW growing pigs according to standard recommendations^72^, with corn, wheat middling, and soybean meal, and containing 13.53 MJ of DE, 164 g of CP (Table 6). Feed allowance was standardized at 2 kg/day of feed, which corresponds to the third quartile of the average feed intake of growing pigs between 45 and 60 kg^71^. The experiment consisted of three consecutive periods. Period 1 (**P1**) was the initial adaptation period (5 days) where all pigs were offered the same feeding allowance (2 kg/day). Period 2 (**P2**) was a 6-day period during which feed restriction was imposed to half of the animals. During P2, half of the pigs [referred to as Normal Feeding (**NF**), 3 LW and 3 CR per replicate] continued to receive the same feed allowance as in P1 (2 kg/day), whereas the other half [referred to as Restricted Feeding (**RF)**, 3 LW and 3 CR per replicate] were fed at 50% of the NF group (1 kg/day). Period 3 (**P3**) constituted the following 14-day period and corresponded to the refeeding period during which all animals were offered a standard feed allowance of 2 kg/day. In each pen, feed was distributed every morning in one meal into a feed dispenser and all pigs had free access to water at all times from a nipple drinker designed to avoid water spillage.

**Table 6:** Composition of feed.

Item	
**Analysed chemical composition (% of DM)**	
Dry matter	88.0 ± 0.4
CP	16.4 ± 0.5
Ash	6.0 ± 0.3
Crude fibre	4.3 ± 0.3
NDF^a^	14.8
Fat	3.8
Starch	42.7
**Energy value (MJ/kg)** ^**b**^	
Gross energy	16.33
DE	13.53

^a^The samples were pooled for the determination of NDF.

^b^Values calculated according to Le Goff and Noblet (2001).

### Measurements

Ambient temperature and relative humidity were continuously recorded (1 measurement every hour) in the room of the closed experimental facilities using a stand-alone USB data logger (EL-USB-2+; DATAQ Instruments, Inc., Akron, OH) located in the centre of the room. Body weight was measured at the beginning of the experiment (d0) and then at the end of each period (i.e. d5, d11 and d26). Backfat thickness (BFT) was measured at the same time as BW except for d0. Backfat thickness measurements were taken ultrasonically (Agroscan, E.C.M., Angouleme, France) at 65 mm from the midline at the point beside the shoulder and at the last rib on each flank.

In order to evaluate the effect of feed restriction and breed on thermoregulation, rectal temperature (RT), skin temperature (ST) and respiratory rate (RR) were measured in all pigs once per period (on days 5, 9 and 25 of the experiment), twice during the day, between 0800 h and 0900 h and between 1100 h and 1500 h. Rectal temperature was recorded with a digital thermometer (Microlife Corp., Paris, France) and ST was measured at the shoulder, mid back (P2 site) using a skin surface thermocouple probe (type K, model 88002K-IEC; Omega Engineering Inc., Stamford, CT) connected to a microprocessor- based handheld thermometer (model HH-21; Omega Engineering Inc.). Respiratory rate (breaths/min) was determined visually by counting flank movements over a period of 1 min, but only for resting pigs.

Blood samples were collected once per period (on days 5, 9 and 25) at 0800 h, before the animals received their meal. Jugular vein blood was obtained (10-mL BD K_2_ EDTA Vacutainers tubes (BD, Franklin Lakes, NJ)) via venepuncture. Blood was centrifuged at 3,400 × g for 10 min at 4 °C to obtain plasma and was then stored at −20 °C or −80 °C depending of the type of analysis to be performed.

Feed samples were taken at the time of diet preparation and were pooled for each week for dry matter determination^73^. Feed refusals were collected daily and when the feed refusal was wet, a sample was collected for dry matter determination.

### Metabolite and Hormone assays

One plasma aliquot was analysed for a routine blood panel consisting of Blood Urea Nitrogen (BUN), creatinine, glucose, total protein, albumin, creatine kinase, amylase, Alanine Transaminase (ALT), Aspartate Transaminase (AST), alkaline phosphatase, γ-glutamyl transferase (GGT), phosphorus and cholesterol. All plasma samples were analysed on an M-Scan II chemistry analyser (Melet Schloesing Laboratoires, Osny, France).

Commercial RIA kits were used to measure plasma concentrations of insulin (CIS Bio International, Gif-sur- Yvette, France), leptin (Millipore Corp., Billerica, MA, USA), and ghrelin (Phoenix Pharmaceuticals, Burlingame, CA, USA).

### Behaviour

Observations were performed for the two first replicates (12 LW, 12 CR) for 24 h during the restriction period, P2 (on day 9 of the experiment), both in NF and RF animals. Pig activities (lateral lying, sternal lying, sitting, standing, eating, drinking) were assessed by an experimenter every 5 min using the scan sampling method. Each experimenter followed pig behaviour during a 3 h shift. The time spent in each posture or activity was expressed at the individual level as a percent of time over the 24 h scanning period.

### Calculations and statistical analysis

Backfat thickness was calculated as the average of BFT measured at rib and shoulder location (right and left). Backfat Gain (BFG) was calculated as the total gain of BFT over the 4 weeks of the experiment divided by initial BFT (in P1) to account for differences in BFT between the two breeds. Average Daily Feed Intake (ADFI, kg/day) was determined for each animal as the difference between feed allowance and refusals. Average daily feed intake, average daily gain (ADG, g/day) and Feed Efficiency (FE, kg of gain per kg of feed) were calculated for each period.

Data were analysed using the MIXED procedure of SAS (SAS Inst., Inc., Cary, NC, USA) including the fixed effects of breed, period, dietary treatment, their interaction and replicate. For BW and ADG, BW at weaning was also included as a fixed effect to take into account environmental maternal effect. For RT, ST and RR, time of measurement was included as a fixed effect. In all statistical analyses using the MIXED procedure of SAS, the repeated measurements option was used to account for animal effect over time with an unstructured covariance structure, except for thermoregulatory variables for which a compound symmetry covariance structure was used, because of no convergence with unstructured covariance structure. Data are reported as least squares means ± SEM and are considered significant if P < 0.05. Residuals from these linear models were used to compute the Pearson correlations between the different hormones and between hormones and performance traits.

Behavioural activities or postures were analysed using a MANOVA including the fixed effect of replicate, breed, treatment and their interactions.

### Ethical approval

All measurements and observations on animals were performed in accordance with the current law on animal experimentation and ethics (#69-2016-1 from the Animal Care and Use Committee of French West Indies and Guyana) and the experimental protocol was approved by the French Ministry of Agriculture and Fisheries (#A971-18-02) under the direction of J. Fleury (INRA-PTEA).

## Data Availability

The datasets generated and analysed during the current study are available from the corresponding author on reasonable request.
